# Intersphincteric resection versus transanal local excision after neoadjuvant therapy for ultra-low rectal cancer: a two-center retrospective propensity-score-adjusted cohort study

**DOI:** 10.3389/fonc.2026.1936588

**Published:** 2026-10-29

**Authors:** Guan-cong Wang, Jia-hong Ye, Yin-cong Guo, Ying Huang

**Affiliations:** 1 Department of Colorectal Surgery, Fujian Medical University Union Hospital, Fuzhou, China; 2 Department of Colorectal and Anal Surgery, Zhangzhou Affiliated Hospital of Fujian Medical University, Zhangzhou, China

**Keywords:** intersphincteric resection, local excision, neoadjuvant chemoradiotherapy, propensity score matching, ultra-low rectal cancer

## Abstract

**Objective:**

To compare the long-term oncological outcomes and postoperative complications between local excision (LE) and intersphincteric resection (ISR) after neoadjuvant chemoradiotherapy (nCRT) in patients with ultra-low rectal cancer (ULRC).

**Methods:**

Data were retrospectively collected from ULRC patients (tumor lower margin ≤5 cm from anal verge) who received nCRT followed by LE or ISR at two hospitals between 2016 and 2025. LE was selectively offered to patients achieving clinical complete response (cCR) or near-complete response (ncCR) after nCRT; ISR was the institutional default for incomplete responders. Propensity score matching (PSM) using 1:2 nearest-neighbor matching with a caliper of 0.2 on the standardized propensity score scale was performed.

**Results:**

A total of 367 patients were enrolled (ISR, n=209; LE, n=158). After PSM, 91 and 56 patients remained, respectively. In the matched cohort, there were 20 deaths, 31 disease-free survival (DFS) events, 17 distant metastases (DM), and 12 local recurrences. No statistically significant differences were observed in overall survival (OS) (HR = 0.699, 95% CI: 0.247–1.977; P = 0.460), DFS (HR = 0.802, 95% CI: 0.369–1.745; P = 0.580), DM (HR = 0.260, 95% CI: 0.059–1.137; P = 0.054), or local recurrence (HR = 1.365, 95% CI: 0.432–4.310; P = 0.590). The LE group had higher rates of overall complications (55.4% vs. 29.7%; P = 0.002) and adapted wound dehiscence (32.1% vs. 13.2%; P = 0.006), predominantly driven by minor complications (Clavien-Dindo I–II: 44.6% vs. 17.6%); major complications were similarly infrequent (10.7% vs. 12.1%). Prophylactic stoma creation was performed in 90.1% of the ISR group versus 7.1% of the LE group (P<0.001). Among 24 LE patients with ypT2 or higher disease, 21 (87.5%) refused recommended salvage surgery.

**Conclusion:**

No statistically significant differences in OS, DFS, DM, or local recurrence were detected between an initial LE and ISR after propensity-score adjustment. However, limited event numbers, residual confounding related to post-neoadjuvant response selection, and incomplete adherence to recommended completion surgery preclude conclusions of oncological equivalence or noninferiority. LE was associated with substantially less initial diversion but more local-excision-defect morbidity under the definitions used. The incidence of postoperative complications was higher after LE, but this was mainly driven by minor complications; surgeons should pay close attention to rectal wound dehiscence.

## 1 Introduction

Ultra-low rectal cancer (ULRC) is typically defined as a tumor with its lower margin ≤5 cm from the anal verge, accounting for approximately one-third of all rectal cancers (1). Intersphincteric resection (ISR) remains one of the most important techniques for achieving sphincter preservation in ULRC (2, 3). With the widespread adoption of neoadjuvant chemoradiotherapy (nCRT) and the advancement of total neoadjuvant therapy (TNT), rates of pathological complete response (pCR) and clinical complete response (cCR) have markedly increased (4, 5). In recent years, local excision (LE) has emerged as another important organ-preserving option for ULRC (6, 7).

These two sphincter-preserving strategies reflect fundamentally different treatment philosophies. ISR pursues maximal oncological radicality through total mesorectal excision (TME) and full nodal staging while maximally preserving anal function, but at the cost of substantial surgical trauma, a high prophylactic stoma rate (exceeding 90%), and impaired anal function (8–12). By contrast, LE preserves the rectal reservoir and sphincter function with better anal function, but offers limited nodal assessment. Whether this intermediate strategy can safely substitute for ISR in routine practice depends on rigorous patient selection, reliable postoperative pathological evaluation, and high patient compliance with potential salvage surgery (13, 14).

However, evidence directly comparing ISR with LE remains limited, as most studies evaluating LE have used TME as a whole—including low anterior rectal resection (LAR), abdominoperineal resection (APR), and ISR—rather than ISR specifically (6, 15–18). These studies have generally shown comparable overall survival (OS) and disease-free survival (DFS), but a higher local recurrence signal in observational cohorts (6, 15). In the modified intention-to-treat analysis of the GRECCAR 2 randomized trial, response-selected LE with protocolized completion TME achieved 5-year oncological outcomes not significantly different from primary TME (local recurrence 7% vs. 7%; OS 84% vs. 82%) (17). A multicenter propensity-matched study from our institutions similarly found no significant differences in OS, DFS, local recurrence, or distant metastasis (DM) between LE and radical resection (13). However, these comparisons grouped ISR with other radical procedures, leaving the specific ISR-versus-LE comparison unresolved. To our knowledge, only one single-center study has directly compared these two strategies, reporting favorable functional outcomes after LE in 51 patients with ypT0–1ycN0 disease (14). However, that study was limited by a small sample size, a single-center design, no systematic assessment of salvage-surgery adherence, and the exclusion of ypT2–3 disease after LE—which may have avoided the real-world challenge of unfavorable pathology, potentially introducing selection bias and limiting generalizability. The specific comparison between ISR and LE in ULRC therefore remains unresolved. Whether LE can safely substitute for ISR in routine practice remains uncertain, particularly in the TNT era where treatment intensity and patient selection vary widely.

This two-center study therefore compared long-term oncological outcomes and postoperative complications between an LE and ISR in ULRC patients after nCRT, using propensity score matching (PSM) to balance baseline characteristics. We specifically evaluated the completion-surgery pathway, including acceptance and refusal rates for salvage surgery, providing more advanced evidence on the clinical trade-offs between these two sphincter-preserving strategies.

## 2 Methods

### 2.1 Study population and ethics

A retrospective analysis was conducted on ULRC patients who received nCRT followed by LE or ISR between January 2016 and January 2025 at Zhangzhou Affiliated Hospital of Fujian Medical University and Fujian Medical University Union Hospital. The study was approved by the Medical Ethics Committees of both institutions (approval numbers: 2026LWB181 for Zhangzhou Affiliated Hospital; 2025KY036 for Union Hospital). The requirement for written informed consent from patients was waived.

### 2.2 Inclusion and exclusion criteria

Inclusion criteria were as follows: (i) histopathologically confirmed rectal adenocarcinoma; (ii) tumor lower margin ≤5 cm from the anal verge; (iii) receipt of preoperative nCRT; (iv) receipt of LE or ISR as the surgical procedure; (v) absence of DM on preoperative assessment; and (vi) complete clinical and follow-up data.

Exclusion criteria were: (i) anal squamous cell carcinoma or other non-adenocarcinoma pathology; (ii) no preoperative nCRT; (iii) absence of downstaging or tumor progression on post-nCRT imaging, rendering surgery impossible (e.g., occurrence of DM, locally unresectable disease, or requirement for palliative surgery); (iv) mid- or high-position rectal cancer with tumor lower margin >5 cm from anal verge; (v) previous history of colorectal cancer surgery or other malignancy; (vi) APR or LAR; and (vii) emergency surgery or concurrent intestinal obstruction/perforation. (**Figure 1**).

**Figure 1:**
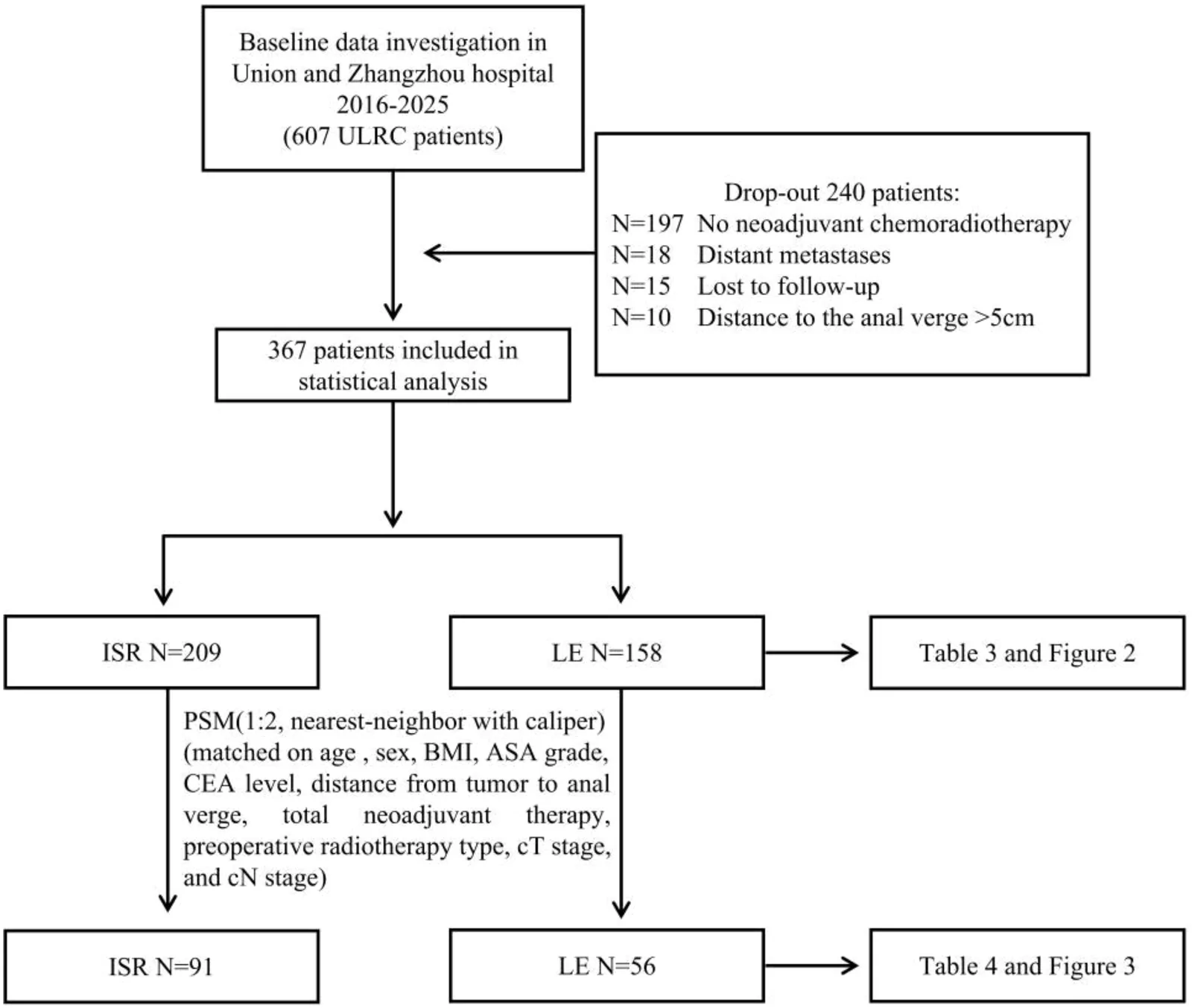
Flowchart of patient selection, group allocation, and propensity score matching (PSM).

### 2.3 nCRT regimen

All patients received either long-course radiotherapy (45–50.4 Gy in 25–28 fractions, 1.8 Gy/fraction) or short-course radiotherapy (25 Gy in 5 fractions, 5 Gy/fraction). After radiotherapy completion, all patients received consolidation chemotherapy with the XELOX regimen (oxaliplatin 130 mg/m² on day 1; capecitabine 1000 mg/m² twice daily on days 1–14, every 3 weeks) or the mFOLFOX6 regimen (oxaliplatin 85 mg/m² on day 1; leucovorin 400 mg/m² on day 1; 5-fluorouracil 400 mg/m² intravenous bolus followed by 2400 mg/m² continuous infusion over 46 hours, every 2 weeks). Standard nCRT consisted of 2–4 consolidation cycles (total systemic cycles 2–4). TNT was defined as additional systemic chemotherapy cycles (up to 6–8 cycles in total) administered either before or after radiotherapy; in practice, this comprised selective induction chemotherapy of 2–4 cycles before radiotherapy plus 4–6 consolidation cycles after radiotherapy, for a total of 6–8 systemic cycles. The exact number of cycles was individualized by the multidisciplinary team (MDT) based on patient tolerance and tumor response. The interval from radiotherapy completion to surgery was 6–12 weeks in the standard nCRT group and 12–24 weeks in the TNT group, reflecting the additional consolidation chemotherapy cycles in the latter (**Supplementary Table S1**).

TNT was selectively applied to patients with a strong desire for sphincter preservation and good performance status to maximize tumor downstaging (19). Of note, TNT was more frequently used in patients who expressed a strong desire for sphincter preservation and had favorable performance status, as it offers the potential for deeper tumor downstaging—a prerequisite for LE eligibility. Thus, TNT use is not merely a prognostic factor but is intrinsically linked to the decision-making process for surgical allocation (LE or ISR). This intertwined relationship was addressed by including TNT as a covariate in the PSM model.

Postoperative adjuvant chemotherapy was not routinely recommended for patients achieving ypT0-1N0. For those with ypT2 or higher, or ypN+, adjuvant chemotherapy was discussed by the MDT and offered, but ultimately deferred to patient preference. This individualized approach was informed by the National Comprehensive Cancer Network (NCCN) guidelines’ recognition that adjuvant chemotherapy decisions after nCRT should be individualized based on pathological stage, prior treatment response, and patient fitness, rather than mandated by a single algorithmic rule (20).

Tumor response was evaluated 4–12 weeks after nCRT completion by digital rectal examination (DRE), endoscopy, and pelvic magnetic resonance imaging (MRI), and, when necessary, positron emission tomography–computed tomography (PET-CT). Clinical response was categorized using a three-tier grading system consistent with the OPRA trial and contemporary ASCRS/ASCO guidance (5, 21, 22):

- cCR: DRE showing no palpable tumor; endoscopy showing a flat, white scar with telangiectasia, without ulceration or nodularity; MRI demonstrating substantial downsizing with no observable residual tumor or fibrosis only (with limited DWI signal), and no suspicious lymph nodes.
- ncCR: DRE showing small, smooth mucosal irregularity; endoscopy showing residual superficial ulceration, small mucosal nodules, or minor mucosal abnormalities with mild persisting erythema; MRI showing obvious downstaging with residual heterogeneous fibrosis or regression of lymph nodes with no malignant enhancement features.
- Incomplete response: Palpable tumor mass on DRE; visible macroscopic tumor on endoscopy; residual tumor mass or persisting extramural disease on MRI.

All MRIs were independently reviewed by two radiologists (at least one attending physician or above), with consensus reached in MDT meetings. The MDT then reassessed the degree of tumor downstaging, patient preference for sphincter preservation, sphincter function, and surgical safety to determine eligibility for LE or ISR.

### 2.4 Treatment allocation logic

#### 2.4.1 For ULRC patients after nCRT, the institutional default recommendation was ISR to ensure oncological radicality

LE was selectively offered only to patients with an extremely strong desire for sphincter preservation, favorable tumor regression (cCR or ncCR), and who voluntarily chose this option after thorough MDT discussion and informed consent. Therefore, patient allocation was based on comprehensive preoperative evaluation rather than individual surgeon preference. Patients allocated to the LE group were fully informed of the potential benefits and risks, particularly the oncological risks associated with postoperative recurrence. It must be acknowledged that selection bias exists between the two groups; accordingly, PSM was employed in this study to balance patient characteristics across groups.

#### 2.4.2 ISR technique

For patients who underwent ISR after nCRT, TME was performed via abdominal dissection, followed by intersphincteric dissection to obtain an adequate distal margin when the distance from the lower tumor margin to the anorectal ring remained <1 cm. ISR was preferentially performed via a completely transabdominal approach for intersphincteric dissection and rectal resection. When an adequate distal or negative margin could not be ensured by the pure transabdominal approach, a hybrid approach was adopted: transabdominal dissection was followed by transanal measurement of the distal tumor margin, and full-thickness division of the mucosa and submucosa was performed to achieve rendezvous with the pelvic dissection. For patients with inadequate resection margins, intraoperative frozen section pathology was performed; if the distal or circumferential resection margin was positive, APR was recommended. All patients received a coloanal end-to-end anastomosis, and a diverting loop ileostomy was routinely constructed unless the patient refused prophylactic stoma creation.

#### 2.4.3 LE technique

LE was performed in patients who achieved substantial tumor downstaging after nCRT. Full-thickness excision was performed with a 1-cm margin from the lesion, completely excising the involved bowel wall muscularis propria and the corresponding partial mesorectal tissue. The defect was then repaired with interrupted full-thickness sutures. The excised specimen was fully spread, pinned on a board, and submitted for pathological examination according to standard protocols. Postoperative pathological evaluation guided further management: If pathology confirmed ypT0–Tis (i.e., pCR or residual carcinoma *in situ* alone), a strict surveillance protocol was recommended. For patients with ypT1 disease, consultation for radical surgery was offered as an optional strategy; patients who opted for surveillance rather than surgery were not classified as having refused radical surgery. For patients with ypT2 or higher disease, salvage radical surgery was strongly recommended; those who declined were explicitly classified as having refused radical surgery. In patients who refused salvage radical surgery, close surveillance was cautiously pursued after full disclosure of the risks of local recurrence and DM.

### 2.5 Outcome measures

Clinical and pathological data were collected for all patients, including: age, sex, body mass index (BMI), American Society of Anesthesiologists (ASA) grade, pretreatment carcinoembryonic antigen (CEA) level, distance to anal verge, clinical T stage (cT), clinical N stage (cN), neoadjuvant regimen (standard nCRT vs. TNT), and radiotherapy type (long-course vs. short-course).

Postoperative histopathological parameters included ypT stage, ypN stage, number of retrieved lymph nodes, lymphovascular invasion (LVI), perineural invasion (PNI), and resection margin status. For the LE group, ypN stage and lymph node yield were not reliably assessable because the partial mesorectal tissue included in the specimen does not permit complete nodal staging, and CRM was not applicable because LE does not produce a total mesorectal specimen. For the ISR group, surgical approach (laparoscopy, open, or robot), ISR approach (complete transabdominal vs. mixed), CRM status, and the number of retrieved lymph nodes were recorded. Quirke grade and detailed margin distances were not systematically recorded in this retrospective cohort; microsatellite instability (MSI) testing was not routinely performed during the study period. Postoperative complications were recorded for all adverse events occurring within 30 days after surgery. Anastomotic leakage (AL) was defined as a defect in the anastomotic wall leading to communication between the intraluminal and extraluminal compartments, or a pelvic abscess adjacent to the anastomosis, confirmed by pelvic CT/MRI or surgical exploration. AL was graded according to the International Study Group of Rectal Cancer (ISREC) criteria (23). For the LE group, wound dehiscence at the excision site was assessed using an adapted three-grade severity classification (adapted from the ISREC framework). Postoperative mortality was defined as death from any cause within 30 days or 90 days after surgery, respectively. Postoperative complications within 30 days were graded per Clavien-Dindo criteria (24). Major complications were defined as Grade ≥III.

The primary outcome was OS, defined as the time from surgery to death from any cause or last follow-up. Secondary outcomes included DFS, time to DM, time to local recurrence, and postoperative complications. DFS was defined as the time from surgery to local recurrence, DM, or death from any cause. Time to DM was defined as the time from surgery to first DM. Time to local recurrence was defined as the time from surgery to recurrence in the pelvis or at the anastomosis.

### 2.6 Follow-up

All patients received regular postoperative follow-up. Considering the higher risk of recurrence in the LE group, follow-up for patients who underwent transanal LE followed the NCCN guidelines (20): proctoscopy combined with endoscopic ultrasound or contrast-enhanced pelvic MRI was performed every 3–6 months during the first 2 years after surgery, and then every 6 months during the subsequent 5 years. For the ISR group, follow-up was conducted every 3 months during the first 2 years, every 6 months during years 3–5, and annually thereafter. Follow-up examinations included physical examination, tumor markers (CEA, CA19-9), colonoscopy, and CT or MRI of the chest, abdomen, and pelvis. Local recurrence and DM were confirmed by imaging and, when necessary, histopathology. The follow-up cut-off date was June 30, 2025.

### 2.7 Statistical analysis

Continuous variables were expressed as mean ± standard deviation and compared using the independent samples t-test. Categorical variables were expressed as frequencies (percentages) and compared using the χ² test or Fisher’s exact test, as appropriate.

To reduce selection bias, propensity score matching (PSM) was performed using R software (version 4.2.0) with the MatchIt package (version 4.5.0). The propensity score was calculated using a logistic regression model that included the following baseline covariates: age, sex, BMI, ASA grade, pretreatment CEA, distance to anal verge, TNT, preoperative radiotherapy type, cT stage, and cN stage. Clinical response category (cCR/ncCR/incomplete) was not included in the propensity model because it was nearly perfectly associated with treatment allocation and would have made matching infeasible. Other post-neoadjuvant pre-surgical response variables (ycT/ycN, residual size, MRI regression, DWI, post-treatment CEA) were not systematically recorded and could not be reliably reconstructed; their omission means that residual confounding by indication cannot be excluded. The treatment variable was coded as LE = 1 and ISR = 0. One-to-two nearest-neighbor matching (ratio = 2) was applied with a caliper of 0.2 on the standardized propensity score scale (caliper = 0.2, std.caliper = TRUE), without replacement (replace = FALSE), in random order (m.order = “random”), and without discarding any patients (discard = “none”). The target estimand was the average treatment effect on the treated (ATT) for LE. Of the 56 matched LE patients, 21 received one ISR match and 35 received two ISR matches, yielding 91 matched ISR controls. All baseline covariates were complete with no missing data; therefore, no imputation was performed. Unmatched patients were excluded from subsequent analyses. Covariate balance after PSM was assessed using standardized mean differences (SMD), with an SMD <0.1 considered acceptable. A Love plot and propensity score overlap plot were generated using the cobalt package (version 4.5.0) to visualize balance improvement (**Supplementary Figures S1**, **S2**). Although P values from the χ² test or t-test are presented in **Table 1**, they were used for reference only and not as the primary evidence of balance.

**Table 1:** Baseline characteristics of patients before and after PSM, n (%).

Characteristic	Subgroup	Before PSM				After PSM			
		ISR group	LE group	SMD	P value	ISR group	LE group	SMD	P value
		(n = 209)	(n = 158)			(n = 91)	(n = 56)		
Sex	Man	111 (53.1)	87 (55.1)	0.04	0.790	50 (54.9)	34 (60.7)	0.12	0.607
	Female	98 (46.9)	71 (44.9)			41 (45.1)	22 (39.3)		
Age (years)	≤50	79 (37.8)	30 (19.0)	0.42	<0.001	24 (26.4)	15 (26.8)	0.01	1.000
	>50	130 (62.2)	128 (81.0)			67 (73.6)	41 (73.2)		
BMI (kg/m2)	≤25	162 (77.5)	115 (72.8)	0.11	0.358	72 (79.1)	44 (78.6)	0.01	1.000
	>25	47 (22.5)	43 (27.2)			19 (20.9)	12 (21.4)		
ASA grade	I	19 (9.1)	31 (19.6)	0.13	0.005	8 (8.8)	7 (12.5)	0.04	0.741
	II	181 (86.6)	116 (73.4)			75 (82.4)	45 (80.4)		
	III	9 (4.3)	11 (7.0)			8 (8.8)	4 (7.1)		
Pretreatment CEA (ng/ml)	<5	147 (70.3)	115 (72.8)	0.05	0.691	63 (69.2)	40 (71.4)	0.05	0.923
	≥5	62 (29.7)	43 (27.2)			28 (30.8)	16 (28.6)		
Distance to anal verge	mm	42.2 ± 8.1	33.0 ± 13.0	0.85	<0.001	41.6 ± 9.0	40.5 ± 10.7	0.11	0.518
Total neoadjuvant therapy	No	178 (85.2)	52 (32.9)	1.07	<0.001	65 (71.4)	38 (67.9)	0.08	0.784
	Yes	31 (14.8)	106 (67.1)			26 (28.6)	18 (32.1)		
Preoperative radiotherapy	Long-course	127 (60.8)	119 (75.3)	0.31	0.005	62 (68.1)	37 (66.1)	0.04	0.938
	Short-course	82 (39.2)	39 (24.7)			29 (31.9)	19 (33.9)		
cT stage	T2	25 (12.0)	42 (26.6)	0.15	0.002	16 (17.6)	11 (19.6)	0.04	0.898
	T3	98 (46.9)	63 (39.9)			49 (53.8)	28 (50.0)		
	T4	86 (41.1)	53 (33.5)			26 (28.6)	17 (30.4)		
cN stage	N0	94 (45.0)	60 (38.0)	0.14	0.215	35 (38.5)	19 (33.9)	0.09	0.706
	N+	115 (55.0)	98 (62.0)			56 (61.5)	37 (66.1)		

PSM, Propensity score matching; ISR, Intersphincteric resection; LE, Local resection; BMI, Body mass index; ASA, American Society of Anesthesiologists; CEA, carcinoembryonic antigen.

Survival curves were plotted using the Kaplan-Meier (K-M) method, and differences between groups were compared using the log-rank test. Matched-set membership was accounted for in standard-error estimation using cluster-robust variance estimation by matched set for Cox models and survey-weighted K-M estimates with matched set as the cluster. Univariate and multivariate Cox proportional hazards regression analyses were performed. Covariates were entered into the multivariable Cox model only if they reached P<0.10 in univariable analysis. Before PSM (38 deaths), TNT and cN stage were entered into the multivariable model. After PSM (20 deaths), a Cox proportional hazards model was fitted using the LR forward stepwise method, which retained ASA grade as the only covariate. A separate Cox model entering all covariates simultaneously (Enter method) was also performed for comparison. The proportional-hazards assumption was tested using Schoenfeld residuals and was not violated. Post-match estimates incorporated MatchIt matching weights and used cluster-robust variance estimation by matched set. All tests were two-sided, and P<0.05 was considered statistically significant. All statistical analyses were conducted using R software (version 4.2.0).

## 3 Results

### 3.1 Patient baseline characteristics

A total of 367 ULRC patients who received nCRT followed by ISR or LE were enrolled in this study, including 209 patients in the ISR group and 158 in the LE group. Before PSM, significant differences were observed between the two groups in several baseline variables: the LE group had a higher proportion of patients aged >50 years (81.0% vs. 62.2%, P<0.001), a shorter distance to anal verge (33.0 ± 13.0 mm vs. 42.2 ± 8.1 mm, P<0.001), a higher rate of TNT administration (67.1% vs. 14.8%, P<0.001), and a higher proportion of long-course radiotherapy (75.3% vs. 60.8%, P = 0.005). More importantly, the LE group had an earlier preoperative cT stage: the proportion of cT2 stage was significantly higher in the LE group than in the ISR group (26.6% vs. 12.0%), while the proportion of cT3/T4 stage was relatively lower (73.4% vs. 88.0%, P = 0.002), indicating that the LE group was more likely to select patients with a relatively earlier tumor stage (cT2).

After PSM, 91 ISR patients and 56 LE patients were successfully matched, totaling 147 patients. Covariate balance was assessed using SMD. Most covariates achieved SMD <0.1, indicating acceptable balance between the two groups. Two variables—sex (SMD = 0.12) and distance to anal verge (SMD = 0.11)—had SMD slightly above the conventional 0.1 threshold (**Table 1**; **Supplementary Figure S1**). These minor imbalances were acknowledged, but no additional sensitivity analysis was performed because the limited number of deaths in the matched cohort (20) precluded further covariate adjustment in the matched analysis. (**Table 1**).

Among the 158 patients in the LE group, postoperative pathology revealed ypT0–T1 in 134 patients (84.8%), ypT2 in 21 (13.3%), and ypT3 in 3 (1.9%). For all 24 patients with ypT2 or higher disease, salvage radical surgery was strongly recommended; three patients accepted and underwent the procedure, while 21 (21/24, 87.5%) refused and opted for intensive surveillance. A patient with ypT1 disease was offered surgical consultation, voluntarily requested radical surgery, and underwent the procedure; this patient was not categorized as refusing salvage surgery. All patients in both groups achieved R0 resection. The LE group showed significantly more pronounced downstaging after nCRT (ypT0–1: 134 patients, 84.8%) compared with the ISR group (ypT0–1: 72 patients, 34.4%; ypT2: 62, 29.7%; ypT3: 68, 32.5%; ypT4: 7, 3.3%). For the ISR group, CRM was negative in all cases, and the mean number of retrieved lymph nodes was 12.0 ± 6.5 before PSM and 11.1 ± 5.9 after PSM. Additional postoperative pathological features—including TRG, LVI, PNI, and resection margin status—are summarized descriptively in **Table 2**. In the ISR group, TRG 0 was observed in 54 patients (25.8%), LVI in 20 (9.6%), and PNI in 23 (11.0%); in the LE group, the corresponding figures were 108 (68.4%), 10 (6.3%), and 17 (10.8%), with no positive margins in either group. However, these variables had considerable missingness in the LE group (TRG: 19.0%; LVI: 25.3%; PNI: 20.9%), primarily reflecting the marked tumor regression after neoadjuvant therapy, which left minimal or no residual tumor for reliable assessment. For the LE group, ypN stage and lymph node yield were not reliably assessable because the partial mesorectal tissue included in the specimen does not permit complete nodal staging, and CRM was not applicable because LE does not produce a total mesorectal specimen. Because of these inherent limitations and the substantial missing data, formal statistical comparisons were not performed for these postoperative variables, and they are presented for descriptive characterization only. (**Table 2**).

**Table 2:** Postoperative pathological characteristics and surgical details of the ISR and LE groups before and after propensity score matching.

Characteristic	Subgroup	Before PSM		After PSM	
		ISR group	LE group	ISR group	LE group
		(n = 209)	(n = 158)	(n = 91)	(n = 56)
ypT stage	T0-1	72 (34.4)	134 (84.8)	37 (40.7)	48 (85.7)
	T2	62 (29.7)	21 (13.3)	24 (26.4)	8 (14.3)
	T3	68 (32.5)	3 (1.9)	29 (31.9)	0 (0.0)
	T4	7 (3.3)	0 (0.0)	1 (1.1)	0 (0.0)
ypN stage	N0	155 (74.1)	—	69 (75.8)	—
	N+	54 (25.8)	—	22 (24.2)	—
Post-neoadjuvant clinical response	cCR	51 (24.4)	110 (69.6)	24 (26.4)	34 (60.7)
	ncCR	67 (32.1)	26 (16.5)	36 (39.6)	11 (19.6)
	Incomplete response	61 (29.2)	6 (3.8)	18 (19.8)	1 (1.8)
	Missing	30 (14.4)	16 (10.1)	13 (14.3)	10 (17.9)
Number of lymph nodes		12.0 ± 6.5	—	11.1 ± 5.9	—
Tumor regression grade (TRG)	TRG 0	54 (25.8)	108 (68.4)	25 (27.5)	37 (66.1)
	TRG 1-3	122 (58.4)	20 (12.7)	63 (69.2)	6 (10.7)
	Missing	33 (15.8)	30 (19.0)	3 (3.3)	13 (23.2)
Lymphovascular invasion (LVI)	No	183 (87.6)	108 (68.4)	84 (92.3)	37 (66.1)
	Yes	20 (9.6)	10 (6.3)	5 (5.5)	7 (12.5)
	Missing	6 (2.9)	40 (25.3)	2 (2.2)	12 (21.4)
Perineural invasion (PNI)	No	181 (86.6)	108 (68.4)	85 (93.4)	37 (66.1)
	Yes	23 (11.0)	17 (10.8)	4 (4.4)	2 (3.6)
	Missing	5 (2.4)	33 (20.9)	2 (2.2)	17 (30.4)
Positive resection margin	Yes	0 (0.0)	0 (0.0)	0 (0.0)	0 (0.0)
ISR approach	Complete transabdominal	178 (85.2)	—	75 (82.4)	—
	Mixed	31 (14.8)	—	16 (17.6)	—
Surgical method	Laparoscopy	165 (78.9)	—	71 (78.0)	—
	Open	34 (16.2)	—	16 (17.5)	—
	Robot	10 (4.7)	—	4 (4.3)	—
Receive radical surgery after LE	No	—	154 (97.5)	—	55 (98.2)
	Yes	—	^a^4 (2.5)	—	1 (1.8)
Refuse radical surgery after LE	No	—	3 (12.5)	—	0 (0.0)
	Yes	—	^b^21 (87.5)	—	8 (100.0)

^a^ Includes 3 patients with ypT2+ disease (2 ypT2, 1 ypT3) and 1 patient with ypT1 disease.

^b^ The refusal rate shown in the table (87.5%) is calculated among ypT2+ patients (21/24).

No statistical comparisons were performed for postoperative variables, due to: (1) these are post-treatment outcomes, not baseline covariates; (2) ypN/lymph node data are not reliably assessable to LE group; and (3) TRG, LVI, and PNI have substantial missing data. Data are shown descriptively.

### 3.2 Survival analysis

#### 3.2.1 Survival analysis before PSM

The median follow-up time before PSM, estimated by the reverse Kaplan-Meier method, was 56.0 months (95% CI: 49.6–62.4) for the overall cohort, 63.0 months (95% CI: 59.9–66.1) for the ISR group, and 34.3 months (95% CI: 23.9–44.7) for the LE group. During follow-up, there were 38 deaths (ISR: 29; LE: 9), 62 DFS events (ISR: 41; LE: 21), 41 DM (ISR: 35; LE: 6), and 25 local recurrences (ISR: 14; LE: 11). The 5-year OS rates were 83.7% for the ISR group and 87.5% for the LE group; the 5-year DFS rates were 78.0% and 87.2%, respectively; and the 5-year cumulative local recurrence-free rates were 91.4% and 91.3%, respectively. Before PSM, K-M survival curves showed no statistically significant differences between the ISR and LE groups in OS (HR = 0.873, 95% CI: 0.352–2.164; P = 0.180), DFS (HR = 0.737, 95% CI: 0.429–1.267; P = 0.260), or local recurrence (HR = 1.271, 95% CI: 0.576–2.802; P = 0.550). However, the ISR group had a significantly higher probability of DM than the LE group (HR = 0.274, 95% CI: 0.115–0.652; P = 0.002). Because the two groups differed substantially in baseline characteristics and neoadjuvant therapy intensity, these curves before PSM are presented for descriptive purposes only and should not be interpreted causally; the higher DM rate in the ISR group, this pattern should not be interpreted causally; it may reflect baseline differences, treatment selection, or reduced statistical power. (**Figure 2**).

**Figure 2:**
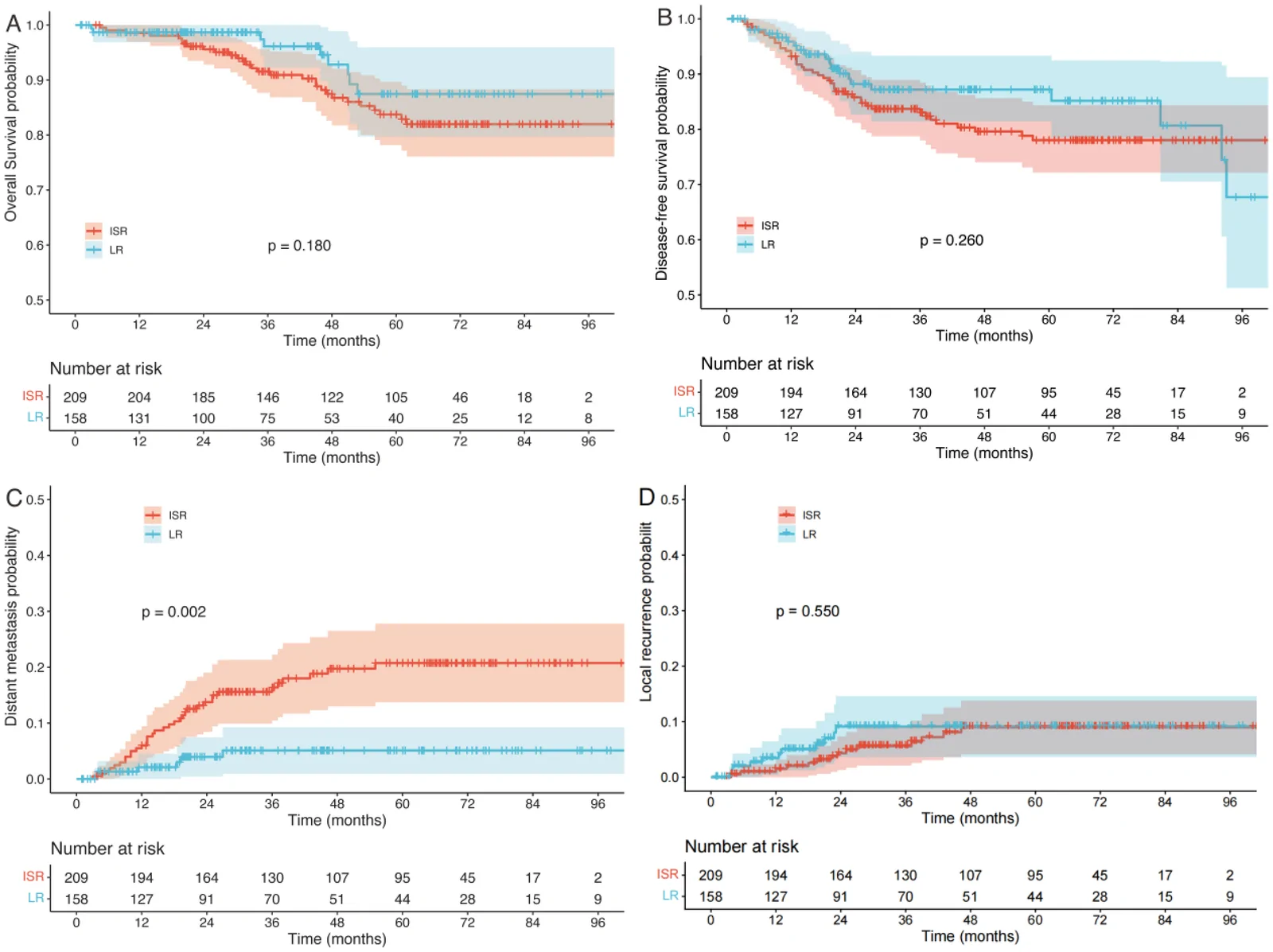
Pre-propensity score matching Kaplan–Meier curves for **(A)** overall survival, **(B)** disease-free survival, **(C)** distant metastasis, and **(D)** local recurrence in patients with ultra-low rectal cancer treated with intersphincteric resection (ISR) versus local excision (LE).

To identify factors associated with OS, univariate and multivariate Cox regression analyses were performed in the cohort before PSM. Multivariable analysis identified TNT (HR = 0.269, 95% CI: 0.082–0.882; P = 0.030) and cN stage (HR = 2.715, 95% CI: 1.284–5.741; P = 0.009) as factors associated with overall survival (**Table 3**). Surgical modality (LE vs. ISR) was not significantly associated with overall survival (HR = 0.873, 95% CI: 0.352–2.164; P = 0.769) in the univariable analysis. (**Table 3**).

**Table 3:** Univariate and multivariate Cox regression analyses for overall survival in ultra-low rectal cancer patients before propensity score matching (n = 367).

Characteristic	Univariate analysis		Multivariate analysis	
	HR (95% CI)	P value	HR (95% CI)	P value
Surgery procedure (LE vs. ISR)	0.873 (0.352–2.164)	0.769		
Sex (Female vs. Man)	1.541 (0.805–2.952)	0.192		
Age (>50 vs. ≤50 years)	0.928 (0.447–1.930)	0.842		
BMI (>25 vs. ≤25 kg/m2)	0.764 (0.331–1.762)	0.528		
ASA (II/III vs. I)	1.578 (0.631–3.943)	0.329		
Pretreatment CEA (≥5 vs. <5 ng/ml)	1.157 (0.580–2.304)	0.679		
Distance to anal verge (mm)	0.977 (0.943–1.013)	0.209		
Total neoadjuvant therapy (Yes vs. No)	0.248 (0.066–0.935)	0.039	0.269 (0.082–0.882)	0.030
Preoperative radiotherapy (Short vs. Long course)	1.282 (0.519–3.168)	0.591		
cT stage (T3/T4 vs. T2)	1.049 (0.640–1.718)	0.850		
cN stage (N+ vs. N0)	2.743 (1.246–6.038)	0.012	2.715 (1.284–5.741)	0.009

BMI, Body mass index; ASA, American Society of Anesthesiologists; CEA, carcinoembryonic antigen.

Multivariable analysis was restricted to covariates with P<0.10 in univariable analysis (TNT and cN stage) to maintain at least 10 events per variable (38 deaths).

#### 3.2.2 Survival analysis after PSM

In the matched cohort, the median follow-up time after PSM, estimated by the reverse Kaplan-Meier method, was 63.0 months (95% CI: 58.6–67.4) for the overall cohort, 64.0 months (95% CI: 60.0–67.7) for the ISR group, and 57.7 months (95% CI: 38.4–77.0) for the LE group. During follow-up, there were 20 deaths (ISR: 15; LE: 5), 31 DFS events (ISR: 21; LE: 10), 17 DM (ISR: 15; LE: 2), and 12 local recurrences (ISR: 7; LE: 5). The 5-year OS rates were 81.2% for the ISR group and 84.9% for the LE group; the 5-year DFS rates were 74.2% and 81.8%, respectively; and the 5-year cumulative local recurrence probabilities were 10.2% for the ISR group and 11.4% for the LE group, respectively. K-M survival curves showed no statistically significant differences between the two groups in OS (HR = 0.699, 95% CI: 0.247–1.977; P = 0.460), DFS (HR = 0.802, 95% CI: 0.369–1.745; P = 0.580), DM (HR = 0.260, 95% CI: 0.059–1.137; P = 0.054), or local recurrence (HR = 1.365, 95% CI: 0.432–4.310; P = 0.590). (**Figure 3**).

**Figure 3:**
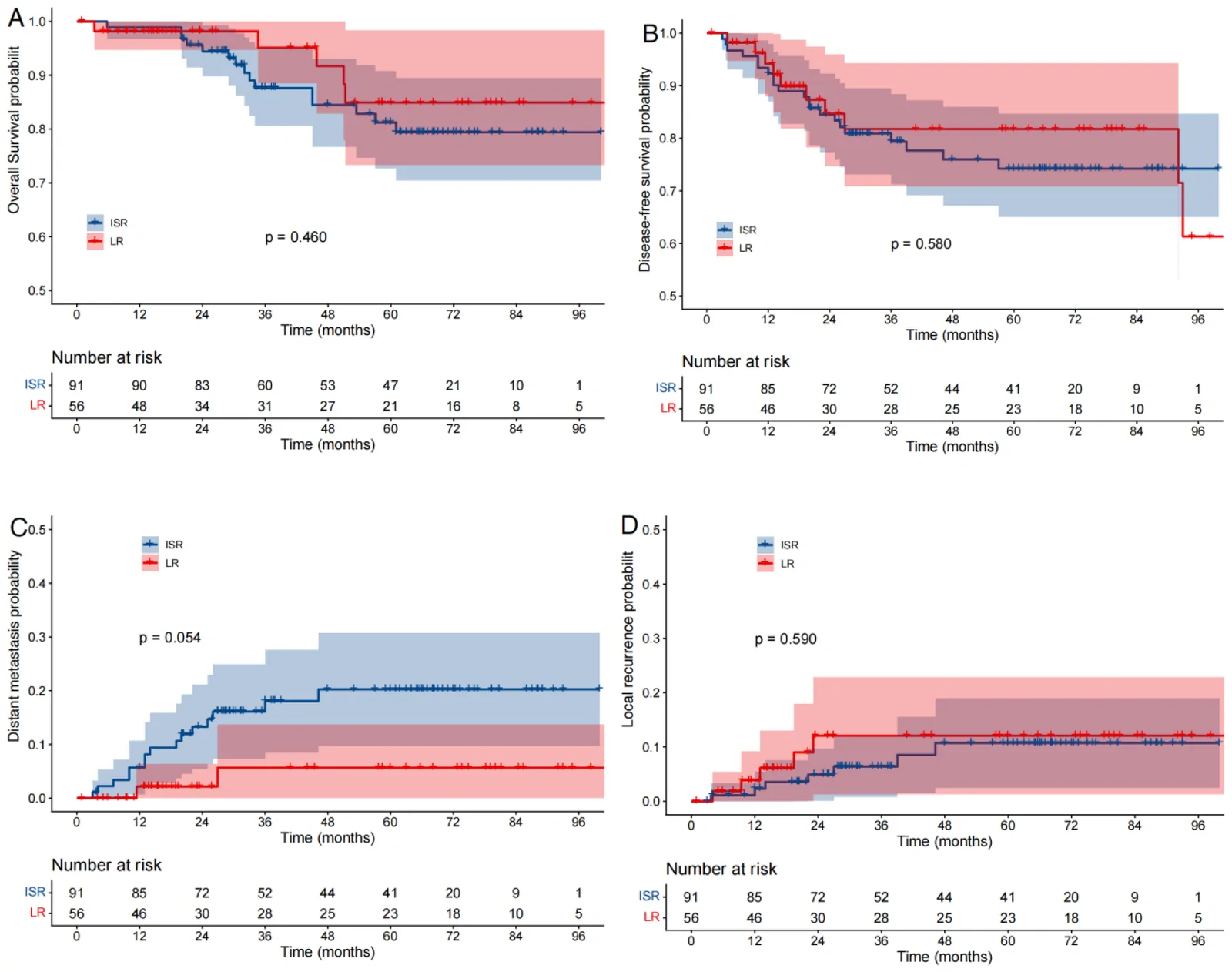
Post-propensity score matching Kaplan–Meier curves for **(A)** overall survival, **(B)** disease-free survival, **(C)** distant metastasis, and **(D)** local recurrence in patients with ultra-low rectal cancer treated with intersphincteric resection (ISR) versus local excision (LE).

In the model entering all covariates simultaneously (Enter method), ASA was associated with OS (HR = 3.529, 95% CI 1.094–11.383; P = 0.035). In the LR forward stepwise model, which retained ASA as the only covariate, ASA remained associated with OS (HR = 3.392, 95% CI 1.242–9.265; P = 0.017). (**Table 4**).

**Table 4:** Univariate and multivariate Cox regression analyses for overall survival in ultra-low rectal cancer patients after propensity score matching (n = 147).

Characteristic	Univariate analysis		Multivariate analysis	
	HR (95% CI)	P value	HR (95% CI)	P value
Surgery procedure (LE vs. ISR)	0.699 (0.247–1.977)	0.499		
Sex (Female vs. Man)	1.834 (0.726–4.635)	0.199		
Age (>50 vs. ≤50 years)	0.770 (0.228–2.604)	0.675		
BMI (>25 vs. ≤25 kg/m2)	0.592 (0.160–2.185)	0.431		
ASA (II/III vs. I)	3.529 (1.094–11.383)	0.035	3.392 (1.242–9.265)	0.017
Pretreatment CEA (≥5 vs. <5 ng/ml)	0.882 (0.314–2.475)	0.812		
Distance to anal verge (mm)	0.966 (0.919–1.015)	0.172		
Total neoadjuvant therapy (Yes vs. No)	0.272 (0.025–2.981)	0.287		
Preoperative radiotherapy (Short vs. Long course)	3.144 (0.336–29.421)	0.315		
cT stage (T3/T4 vs. T2)	0.787 (0.405–1.529)	0.480		
cN stage (N+ vs. N0)	2.350 (0.788–7.009)	0.125		

BMI, Body mass index; ASA, American Society of Anesthesiologists; CEA, carcinoembryonic antigen.

Multivariable analysis was restricted to covariates with P<0.10 in univariable analysis (ASA) to maintain at least 10 events per variable (20 deaths).

Univariable model: all covariates entered simultaneously (Enter method). Multivariable model: LR forward stepwise method, retaining ASA grade only.

### 3.3 Postoperative complications

Because of substantial baseline differences between the two groups before PSM (e.g., tumor stage, nCRT intensity), postoperative complications were compared in the cohort after PSM as the primary analysis, to reduce selection bias and avoid confounding by baseline differences. Before PSM, the overall complication rate was 24.9% in the ISR group versus 47.5% in the LE group (P<0.001); however, these data are presented for descriptive purposes only, as the two groups were not balanced before matching, and the comparison should not be interpreted causally. After PSM, there was no postoperative 30-day or 90-day mortality in either group. The LE group had a significantly higher rate of overall complications than the ISR group (55.4% vs. 29.7%, P = 0.002), with a higher incidence of adapted wound dehiscence in the LE group (32.1% vs. 13.2% for AL in the ISR group, P = 0.006). According to the adapted three-grade severity classification, the LE group had 14 cases (25.0%) of adapted grade B and 4 cases (7.1%) of adapted grade C wound dehiscence, whereas the ISR group had 12 cases (13.2%) of grade B and no grade C AL (P = 0.005 for grade distribution). When stratified by severity using the total cohort as the denominator, the overall distribution across Clavien-Dindo categories (no complication, minor, major) differed significantly between the two groups (P = 0.002). This difference was driven primarily by minor complications (Clavien-Dindo I–II), which were substantially more frequent after LE (25/56, 44.6%) than after ISR (16/91, 17.6%). In contrast, major complications (Clavien-Dindo III–V) were similarly infrequent in both groups (LE: 6/56, 10.7%; ISR: 11/91, 12.1%). The higher overall complication rate in the LE group was therefore predominantly driven by minor complications. In addition, the ISR group had a significantly higher rate of prophylactic stoma (90.1% vs. 7.1%, P<0.001). Bleeding occurred in 4 LE patients (7.1%) from the excision site/wound bed, whereas no anastomotic bleeding occurred in the ISR group (P = 0.020). No statistically significant differences were observed between the two groups in other complications, such as anastomotic or pelvic infection, pneumonia, chylous leakage, urinary retention, readmission, or reoperation rate (all P>0.05). (**Table 5**).

**Table 5:** Postoperative complications before and after propensity score matching.

Complications	Before PSM			After PSM		
	ISR group	LE group	P value	ISR group	LE group	P value
	(n = 209)	(n = 158)		(n = 91)	(n = 56)	
Overall complications	52 (24.9)	75 (47.5)	<0.001	27 (29.7)	31 (55.4)	0.002
Clavien-Dindo classification			<0.001			0.002^†^
- No	157 (75.1)	83 (52.5)		64 (70.3)	25 (44.6)	
- I-II (Minor)	36 (17.2)	60 (38.0)		16 (17.6)	25 (44.6)	
- III-V (Major)	16 (7.7)	15 (9.5)		11 (12.1)	6 (10.7)	
^*^Anastomotic leakage (ISR)/Adapted wound dehiscence (LE)			<0.001			0.006
- Anastomotic leakage (ISR)	21 (10.0)	—		12 (13.2)	—	
- Adapted wound dehiscence (LE)	—	36 (22.8)		—	18 (32.1)	
Anastomotic leakage/Adapted wound dehiscence grade			<0.001			0.005
- Grade A	0 (0.0)	0 (0.0)		0 (0.0)	0 (0.0)	
- Grade B	21 (10.0)	28 (17.7)		12 (13.2)	14 (25.0)	
- Grade C	0 (0.0)	8 (5.1)		0 (0.0)	4 (7.1)	
Anastomotic or pelvic infection	26 (12.4)	32 (20.3)	0.042	15 (16.5)	13 (23.2)	0.313
Bleeding			0.042			0.020
- Anastomotic bleeding	1 (0.5)	—		0 (0.0)	—	
- Wound bleeding	—	8 (5.1)		—	4 (7.1)	
Diverting stoma	186 (89.0)	8 (5.1)	<0.001	82 (90.1)	4 (7.1)	<0.001
Pneumonia	8 (3.8)	1 (0.6)	0.084	5 (5.5)	0 (0.0)	0.157
Chylous leakage	4 (1.9)	0 (0.0)	0.137	3 (3.3)	0 (0.0)	0.288
Urinary retention	4 (1.9)	0 (0.0)	0.137	3 (3.3)	0 (0.0)	0.288
Readmission	7 (3.3)	10 (6.3)	0.179	7 (7.7)	7 (12.5)	0.335
Reoperation	7 (3.3)	15 (9.5)	0.014	2 (2.2)	6 (10.7)	0.054

Postoperative 30-day and 90-day mortality: 0% in both groups.

^†^ P values for the Clavien-Dindo classification (before and after PSM) refer to the overall distribution across the three categories (no complication, minor, major).

^*^ For the LE group, wound dehiscence at the excision site was assessed using an adapted three-grade severity classification, adapted from the International Study Group of Rectal Cancer (ISREC) framework, because LE does not create a colorectal or coloanal anastomosis.

## 4 Discussion

In this multicenter retrospective study, a PSM analysis was used to compare long-term oncological outcomes and postoperative complications between an LE and ISR in ULRC patients after nCRT. The principal findings were as follows: (1) after PSM, no statistically significant differences were observed between the two groups in OS, DFS, DM, or local recurrence; (2) the LE group had significantly higher rates of overall complications and adapted wound dehiscence, whereas ISR was associated with a markedly higher prophylactic stoma rate (90.1% vs. 7.1%); and (3) among 24 patients with ypT2 or higher disease, 21 (87.5%) refused the recommended salvage surgery. Before PSM, the ISR group had a significantly higher probability of DM (HR = 0.274, 95% CI: 0.115–0.652; P = 0.002). After PSM, the point estimate was similar (HR = 0.260, 95% CI: 0.059–1.137), but the confidence interval widened and the difference was no longer statistically significant (P = 0.054). In the matched cohort, no statistically significant difference in OS was observed between the LE and ISR groups (HR = 0.699, 95% CI: 0.247–1.977; P = 0.460), with 5-year OS rates of 84.9% and 81.2%, respectively. These findings are consistent with previous evidence: the GRECCAR 2 randomized trial reported 5-year OS of 84% after response-selected LE with protocolized completion TME versus 82% after primary TME (adjusted HR 0.92, 95% CI 0.38–2.22) (17), and a National Cancer Database propensity-matched analysis found no significant OS difference between LE and proctectomy among ypT0–1 patients (7). Similar results were reported in a multicenter propensity-matched study from our institutions (13) and in pooled meta-analyses, although the latter are limited by the predominantly observational nature of the included studies (6, 15). However, the wide confidence interval in our analysis (0.247–1.977) reflects the limited number of deaths (20 in the matched cohort) and precludes any conclusion of equivalence or non-inferiority. The apparent survival similarity should therefore be interpreted as hypothesis-generating rather than confirmatory, and prospective validation with adequate statistical power remains necessary.

For recurrence outcomes, meta-analyses have reported a higher local recurrence signal after LE in observational cohorts (OR 1.82, 95% CI 1.19–2.77), although this difference was not significant in the randomized-trial subgroup (6, 15). In the present study, no significant differences were observed in DFS (HR = 0.802, 95% CI: 0.369–1.745; P = 0.580), local recurrence (P = 0.590), or DM (HR = 0.260, 95% CI: 0.059–1.137; P = 0.054). Two factors may explain the absence of a local recurrence disadvantage in our cohort. First, the completeness of LE may be important: in our center, LE was performed using full-thickness excision with partial mesorectal excision and a 1-cm margin, a more extensive technique than conventional LE, similar to that adopted in the transanal endoscopic microsurgery group of the RCT by Lezoche et al., which reported a local recurrence rate of only 8% (18). Second, patient selection has a decisive impact: 84.8% of LE patients in our cohort achieved ypT0–1 after nCRT, a subgroup with a nodal metastasis risk below 10% and a lower risk of local recurrence than ypT2 or higher (25, 26). In this cohort, no statistically significant difference in local recurrence was observed between LE and ISR. However, these findings should not be interpreted as evidence that LE with completion surgery preserves local control, because completion surgery was accepted by only 3 of 24 patients (12.5%) with ypT2 or higher disease. The oncological adequacy of an LE-first strategy with protocolized completion surgery therefore cannot be assessed from these data.

Unlike most previous studies, the LE group in the present study had a significantly higher rate of overall complications than the ISR group (55.4% vs. 29.7%, P = 0.002), whereas previous RCTs reported complication rates of only 6–12% after LE (17, 18); a recent study of LE after TNT reported a similar complication profile, suggesting that LE after TNT is not risk-free (27). Several factors may explain the higher adapted wound complication rate in the LE group. First, TNT use was more frequent in this group (67.1% vs. 14.8%), and TNT has been associated with impaired wound healing and increased anastomotic complications (28, 29), possibly through radiation-induced fibrosis and microvascular damage (30). Second, full-thickness excision combined with partial mesorectal excision created a relatively large tissue defect requiring suture repair, with less favorable tension and blood supply, and a residual cavity susceptible to pelvic infection. Third, prophylactic diversion was routinely performed in the ISR group (90.1%) but in only 7.1% of LE patients; consequently, when structural failure occurred in the LE group, its clinical consequences were more severe, as reflected in the higher rate of Grade C events (7.1% vs. 0%) and a trend toward more reoperations (10.7% vs. 2.2%, P = 0.054). However, because diversion was almost deterministically linked to procedure type, its independent contribution cannot be reliably estimated; however, because diversion was almost deterministically linked to procedure type, its independent contribution cannot be reliably estimated in this cohort. We therefore hypothesize that the higher rate of severe adapted wound complications in the LE group may reflect the more severe clinical consequences of leakage in the absence of a protective stoma, but this remains speculative and cannot be distinguished from a higher underlying rate of structural failure. This explanation should be regarded as hypothesis-generating only. Notably, when complications were stratified by severity, major complications (Clavien-Dindo III–V) were similarly infrequent in both groups (10.7% after LE vs. 12.1% after ISR), whereas minor complications (Clavien-Dindo I–II) were substantially more common after LE (44.6% vs. 17.6%). This indicates that the higher overall complication rate in the LE group was predominantly driven by minor complications, most of which were adapted Grade B wound dehiscence amenable to conservative management, whereas major complications after ISR reflect the greater technical complexity and potential severity of this procedure. These observations suggest that for LE patients who underwent full-thickness excision with partial mesorectal excision, selective use of a prophylactic stoma may be considered when the extent of resection is large and anastomotic tension is high, although this hypothesis requires prospective evaluation.

Watch and Wait (W&W) and LE are both important organ-preserving strategies after nCRT. The core challenge of W&W lies in the discordance between clinical and pathological response: 5–46% of patients with cCR still harbor residual tumor (31), and the International W&W Database reported a local regrowth rate of approximately 25% (32). LE, by contrast, provides pathological confirmation of the ypT stage and thus a more objective basis for subsequent decision-making, although it carries its own risk of complications, as observed in the present study. More critically, once additional radical surgery is indicated, a substantial proportion of patients refuse the recommended treatment: 21 of 24 patients (87.5%) with ypT2 or higher disease in our cohort declined salvage surgery, a rate considerably higher than the 45% reported by Peltrini et al. (33). For ypT1 disease, salvage surgery was offered as a consultation rather than a strong recommendation, consistent with the low risk of nodal metastasis in this subgroup. These findings highlight the importance of preoperative counseling regarding the probability and consequences of needing salvage surgery, enhanced MDT communication for patients with unfavorable pathology, and stringent surveillance for those who decline salvage surgery.

Several limitations of this study should be acknowledged. First and most importantly, this is a retrospective, response-selected observational study, not a randomized trial. LE was selectively offered to patients who achieved favorable tumor downstaging (cCR/ncCR) after nCRT, creating a fundamental risk of confounding by indication that cannot be fully eliminated by PSM. Because surgical allocation occurred after assessment of response to neoadjuvant therapy, several post-neoadjuvant but presurgical response variables (cCR/ncCR category, ycT/ycN, residual lesion size, MRI regression, DWI signals, post-treatment CEA) were potential confounders of the LE-versus-ISR comparison. These variables could not be incorporated reliably into the propensity model: the clinical response category was nearly perfectly associated with treatment allocation and would have made matching infeasible, while the remaining response measures were not systematically recorded and could not be reliably reconstructed retrospectively. Substantial residual confounding by indication therefore remains, and the matched analysis should be interpreted as an association rather than a causal effect and cannot establish non-inferiority or equivalence between LE and ISR. Second, the limited number of oncological events in the matched cohort (20 deaths, 31 DFS events, 17 DM, 12 local recurrences) constrains statistical precision, with wide confidence intervals around the effect estimates (e.g., OS: HR 0.699, 95% CI 0.247–1.977). The absence of statistical significance (P>0.05) should not be overinterpreted as evidence of comparable outcomes or non-inferiority. Follow-up duration differed substantially before PSM, with a median of 63.0 months (95% CI: 59.9–66.1) in the ISR group versus 34.3 months (95% CI: 23.9–44.7) in the LE group. This difference was substantially reduced after PSM, with a median follow-up of 64.0 months (95% CI: 60.0–67.7) in the matched ISR group versus 57.7 months (95% CI: 38.4–77.0) in the matched LE group. Nonetheless, the confidence interval for the matched LE group remained wide, reflecting heterogeneity in follow-up duration, and a considerable proportion of LE patients still had less than 5 years of follow-up. Therefore, although the matched analysis provides a more balanced comparison than the pre-matching analysis, the 5-year outcome estimates for the LE group should still be interpreted with caution. Third, the near-complete collinearity between surgical procedure and prophylactic stoma creation (90.1% in ISR vs. 7.1% in LE) precludes reliable estimation of diversion’s effect on anastomotic/wound complications. Fourth, this study lacks systematic assessment of functional outcomes and quality of life using validated instruments (LARS, Wexner, FIQL, EORTC QoL), and stoma reversal rates, permanent stoma rates, and stoma-related reinterventions were not reliably captured due to outside-hospital reversals and incomplete follow-up. We have not attempted retrospective reconstruction of these outcomes. Fifth, unlike ISR which provides complete mesorectal specimens with full nodal evaluation, LE yields only a local full-thickness specimen, precluding pathological nodal status (pN) assessment and detailed evaluation of risk factors such as LVI, PNI, TRG, and MSI in the LE group; we therefore used pre-treatment cN status for PSM. Sixth, the two centers contributed unequal proportions of patients to the two treatment groups, and TNT adoption evolved substantially during the 2016–2025 study period. We did not perform center- or calendar-era-adjusted sensitivity analyses because the limited number of matched patients and oncological events would have made such models unstable. These factors therefore represent potential residual confounders that could not be fully addressed in this retrospective cohort, and the results should be interpreted with this limitation in mind.Seventh, the salvage surgery pathway was incompletely captured: only 3 of 24 ypT2+ patients (12.5%) accepted completion surgery, while 21 (87.5%) declined, limiting evaluation of the full LE pathway. Finally, while our institution previously reported superior functional outcomes after LE in a smaller single-center cohort of 106 patients (51 LE and 55 ISR) (14), that entire cohort is included within the present dataset. Those functional findings cannot be extrapolated to the current population, as functional outcomes were not measured in the present study. These limitations should be considered when interpreting our findings, and future prospective studies with prespecified non-inferiority margins, standardized response assessment, validated functional instruments, and systematic capture of stoma and salvage outcomes are urgently needed.

## 5 Conclusion

In this retrospective, response-selected cohort, no statistically significant differences in OS, DFS, DM, or local recurrence were detected between an initial LE strategy and ISR after propensity-score adjustment. However, limited event numbers, residual confounding related to post-neoadjuvant response selection, and incomplete adherence to recommended completion surgery preclude conclusions of oncological equivalence or noninferiority. LE was associated with substantially less initial diversion but more local-excision-defect morbidity under the definitions used. Although major complications were similarly infrequent in both groups, surgeons should pay close attention to rectal wound dehiscence, particularly in the absence of a prophylactic stoma. Prospective evaluation with standardized response assessment, systematic capture of completion-TME adherence, long-term stoma outcomes, and validated functional measures is warranted.

## Data Availability

The raw data supporting the conclusions of this article will be made available by the authors, without undue reservation.
