# Supplementary material for: Intersphincteric resection versus transanal local excision after neoadjuvant therapy for ultra-low rectal cancer: a two-center retrospective propensity-score-adjusted cohort study
Source: Front Oncol. 2026 Oct 29;16:1936588. doi: 10.3389/fonc.2026.1936588 (PMC13754641; doi:10.3389/fonc.2026.1936588)

Propensity Score Distribution Before and After Matching

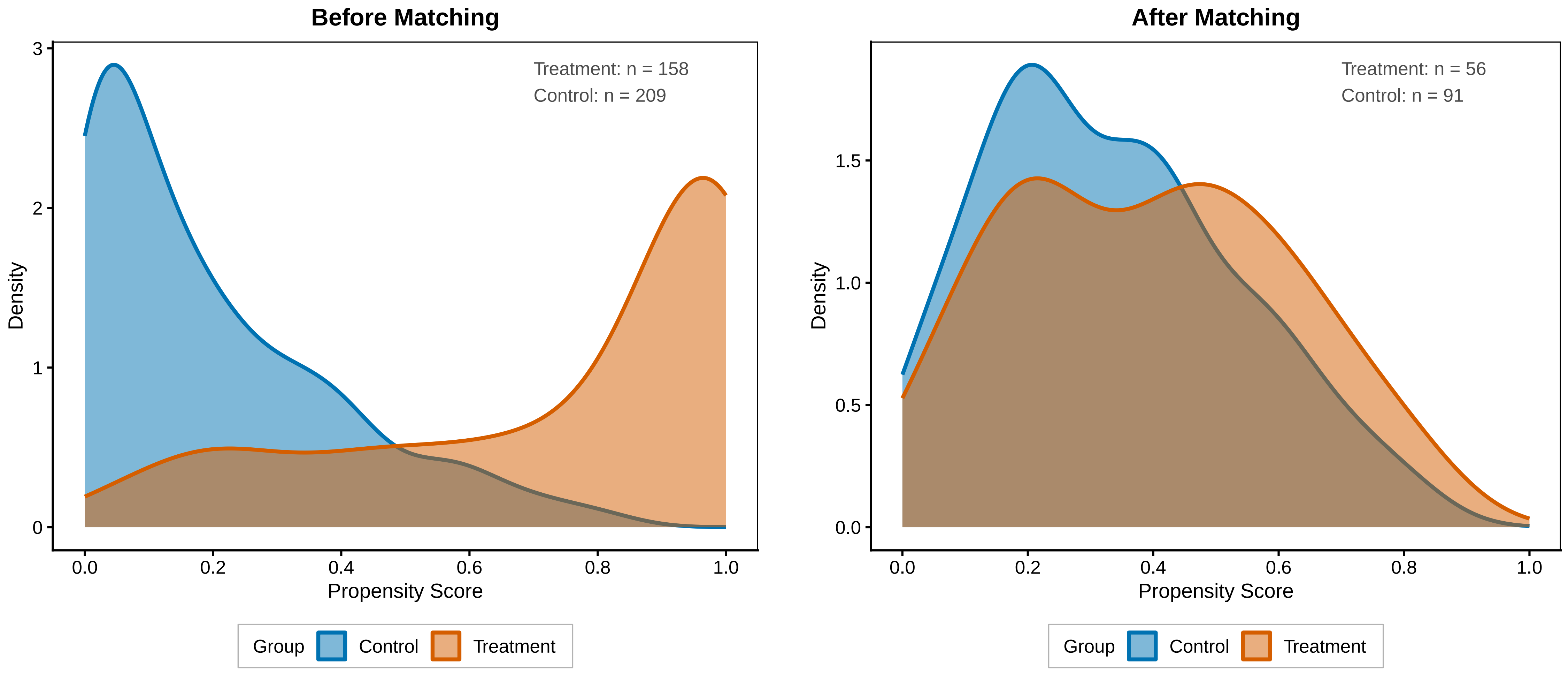

Supplement: Supplementary file 1 [file DataSheet1.pdf]
