# Supplementary material for: Intersphincteric resection versus transanal local excision after neoadjuvant therapy for ultra-low rectal cancer: a two-center retrospective propensity-score-adjusted cohort study
Source: Front Oncol. 2026 Oct 29;16:1936588. doi: 10.3389/fonc.2026.1936588 (PMC13754641; doi:10.3389/fonc.2026.1936588)
Supplement: Supplementary file 2 [file DataSheet2.pdf]

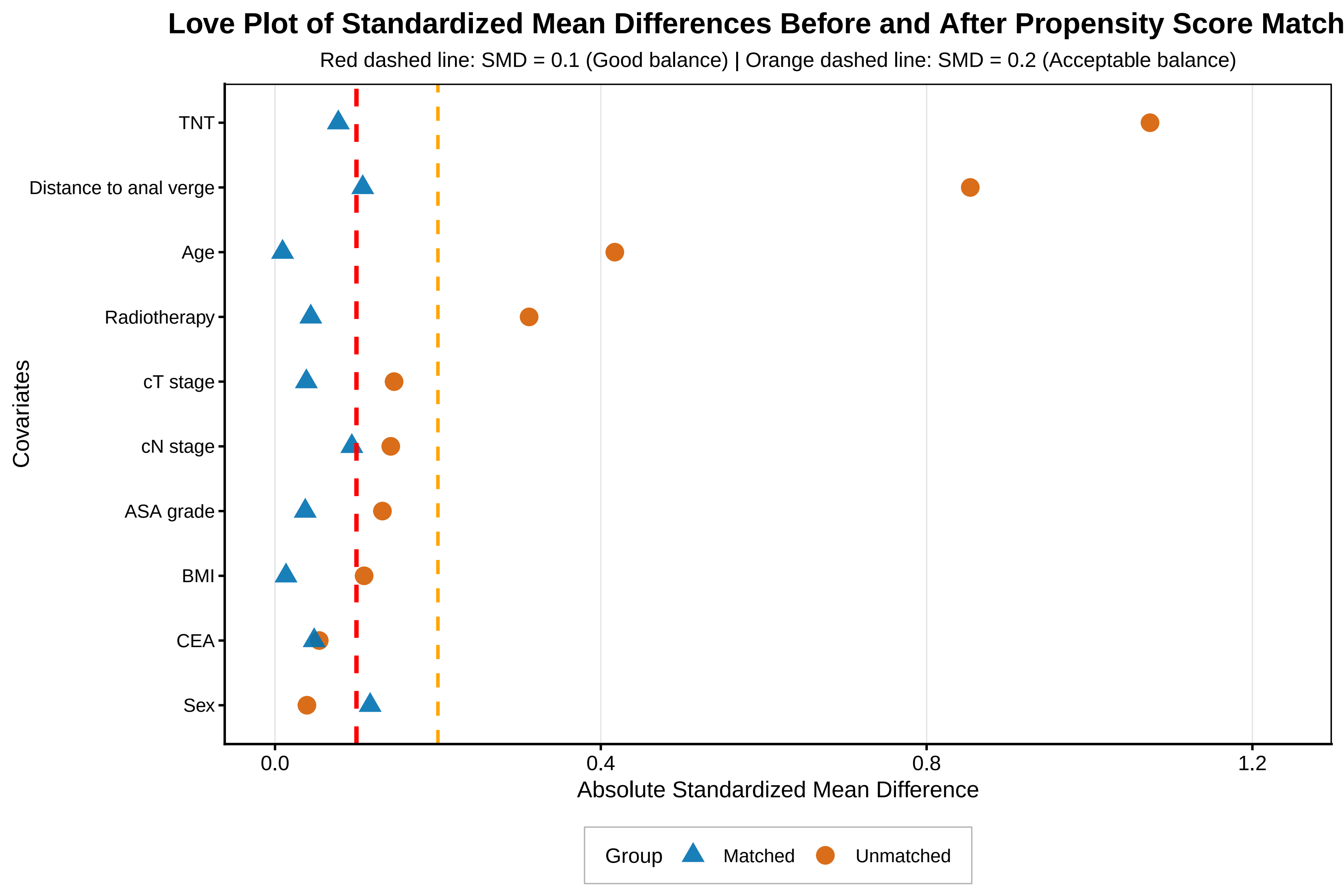

Love Plot of Standardized Mean Differences Before and After Propensity Score Match
Red dashed line: SMD = 0.1 (Good balance) | Orange dashed line: SMD = 0.2 (Acceptable balance)
Covariates
TNT
Distance to anal verge
Age
Radiotherapy
cT stage
cN stage
ASA grade
BMI
CEA
Sex
0.0
0.4
0.8
1.2
Absolute Standardized Mean Difference
Group
Matched
Unmatched
